# Effect of Poly-γ-glutamic Acid on Kinetics of Enamel Demineralization in vitro at pH 4.0, 4.5 and 5.0

**DOI:** 10.1007/s00223-026-01545-3

**Published:** 2026-05-21

**Authors:** Zeeshan Qamar, Zubaidah Binti Haji Abdul Rahim, Robert Hill, Hooi Pin Chew, Paul Anderson

**Affiliations:** 1 Department of O&MFS and Diagnostic Sciences, College of Medicine and Dentistry, Riyadh Elm University, Riyadh, Saudi Arabia; 2 Department of Oral Biology and Biomedical Sciences, Faculty of Dentistry, University Malaya, Kuala Lumpur, Malaysia; 3 Centre for Oral Bioengineering, Institute of Dentistry, Queen Mary University of London, London, UK; 4 Department of Restorative Sciences, Division of Operative Dentistry, School of Dentistry, University of Minnesota, Minneapolis, USA; 5 Centre for Oral Bioengineering, Dental Physical Sciences Unit, Institute of Dentistry, Queen Mary University of London, Mile End Road, London, E1 4NS UK

**Keywords:** Poly-γ-glutamic acid, Statherin, Demineralization, Ion selective electrode, Cross-sectional microhardness

## Abstract

The aim was to use ion selective electrodes and microhardness studies to measure the effects of poly gamma glutamic acid (PGGA) on the demineralization kinetics of dental enamel subjected to simulated cariogenic continuous challenges at pH values of 4.0, 4.5, and 5.0. A range of concentrations of microbially produced PGGA (1% and 2%) and NaF (0.01%, 0.1%, and 0.5%) was prepared. Sodium fluoride (NaF) was used as positive control and phosphate buffer saline (PBS) solution as negative control. Buffered acetic acid solutions (0.1 M) at pH 4.0, 4.5, and 5.0 were used as demineralizing solutions. Fifty-four orthodontically extracted teeth (each varnished leaving a window of 2 × 2 mm) were pre-treated for 24 h with either the respective concentrations of NaF, or PGGA, and then immersed in demineralizing solutions. The calcium ions released from the PGGA and the NaF pretreated enamel in the demineralizing solutions at different pHs were continuously measured using Ca^2+^ Ion Selective Electrodes (Ca^2+^ ISEs). Subsequently, the teeth were subjected to cross-sectional microhardness (CSMH) to determine the mineral concentration as a function of depth. Ca^2+^ ISE data showed enamel pre-treated with the different concentrations PGGA inhibited demineralization at all pHs, as was seen for the positive controls. Further, microhardness data on the same specimens showed that PGGA and the positive controls had not only protected the surface but also within the body of the lesions. PGGA, similarly to NaF, reduces enamel demineralization under artificial lesion formation conditions, not just at the surface but also within the body of the lesion.

**Supplementary Information:**

The online version contains supplementary material available at 10.1007/s00223-026-01545-3.

## Introduction

The *modus operandi* of salivary proteins like statherin in reducing the kinetics of calcium hydroxyapatite (HAp) and enamel dissolution during simulated caries challenges is thought to be associated with interaction of its two N-terminal glutamic acid residues (residues 4 and 5) with hydroxyapatite surfaces [1]. Statherin has a high affinity for the HAp mineral component of dental hard tissues [2]. Further, this strong affinity for the surfaces of enamel means statherin plays a major role in the formation of the protein layer on the tooth surface, the “acquired enamel pellicle” [3].

**Fig. 1:**
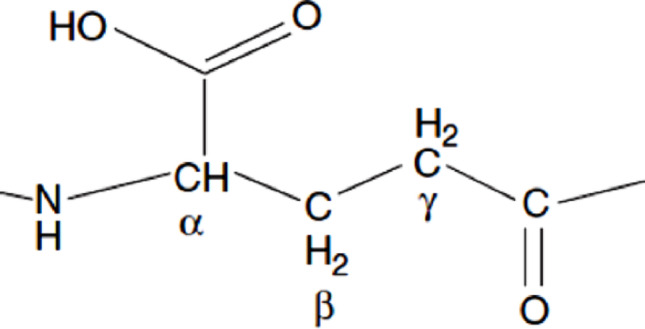
Poly-γ-glutamic acid structure unit peptide structure

Poly-γ-glutamic acid (PGGA) is a naturally occurring polypeptide composed entirely of gamma linked glutamic acid residues, composed of both the D- and L- forms of glutamic acid amino acids connected between the α-amino group of one glutamic residue and the γ-carboxyl group of the adjacent glutamic acid residue (Fig. 1).

This type of linkage has strong resistance against for example salivary proteases cleaving these particular amide bonds. PGGA is hydrophilic and negatively charged at pH above 2.2. In historical order; in 1906, Sawamura first described the Japanese traditional food ‘Natto’, a mixture of PGGA and fructans formed by the fermentation of soya beans [4]; in 1937 Ivanovics discovered PGGA is a component of the *Bacillus anthracis* capsule [5]; in 1942 Bovarnick reported that PGGA is accumulated as an end product of fermentation in a culture broth of *Bacillus Subtilis* [6]. PGGA exists in various forms; broadly classified as; a free acid form; and a salt form. The salt form of PGGA contains either Na^+^, Mg^2+^, K^+^, NH_4_^+^ or Ca^2+^. PGGA has recently been reported to have many health-care benefits [7], including anti-cariogenic effects. Microbially derived PGGA generally has a relatively high molecular weight (Mw ~ 10^5^ – 8 × 10^6^ Da), although that of synthetically produced PGGA can be much lower [8, 9].

Under caries-like pH conditions statherin, and statherin like peptides inhibit in vitro demineralization of HAp, often in the form of HAp pellets models of enamel [10–12]. A key amino acid in these peptides is glutamic acid, which has a carboxyl group side chain which can bind to calcium ions. As glutamic acid is critical to the cariostatic function of the salivary protein statherin, it has been suggested that PGGA may also have similar cariostatic function [13, 14]. A more recent study in which enamel was sequentially exposed to pHs of 4.0, 5.0, and 6.0 concluded that PGGA can inhibit and promote remineralization of human dental enamel, which could be attributed to its quality of coating, and presence of − COO − group [15]. Recently, Parati et al. demonstrated the inhibition activity of PGGA in HAp compressed powder systems in simulated caries conditions [16]. Therefore, the aim of this study was to measure the effect of PGGA on enamel demineralization kinetics at a range of cariogenic pHs (4.0, 4.5, and 5.0) at constant pH value using Ca^2+^ release rates (R_Ca_^2+^) as a proxy for demineralization rate measured with calcium Ion Selective Electrodes (Ca^2+^ ISE) [17]. Further, cross-sectional microhardness (CSMH) measurements under similar conditions were used to corroborate the Ca^2+^ ISE results, specifically to investigate the capability of PGGA to transport calcium ions during artificial carious lesions formation. Sodium fluoride (NaF) solutions were used as positive controls.

## Materials & Methods

### Chemicals and Biological Samples

Microbially produced PGGA was obtained from Nippon PolyGlu (Japan). Samples were weighed accordingly to prepare two concentrations, 1% (w/v) and 2% (w/v), in deionized water. NaF solutions were prepared at 0.01% (w/v), 0.1% (w/v), and 0.5% (w/v) in deionized water.

Phosphate Buffer Saline (PBS) solution was prepared according to the manufacturer’s instructions (Thermo Fischer, U.K) made up with deionized water. Three 1.0 L stock solutions of 0.1 M acetic acid were prepared, adjusted to pH 4.0, 4.5, or 5.0 by adding drops of 1 M NaOH.

Ethical approval was obtained from the Ethics Committee of the Faculty of Dentistry, University of Malaya, Kuala Lumpur, Malaysia [Ethics Committee Reference Number: DF OB1504/0067(P)]. Fifty-four extracted sound premolars were obtained after taking written consent from patients at the Ziauddin University Hospital, Karachi, Pakistan and were stored in PBS buffered solutions. Teeth with i) coronal caries or ii) with root caries or iii) tooth wear were excluded.

### Determination of the Kinetics of Enamel Demineralization using Real-time Ca^2+^ ISE

As demineralization progresses, calcium ions are released into the demineralizing solution. The resulting increase in calcium ion concentration with time can be measured using calcium ion selective electrodes (Ca^2+^ ISE) and an associated analyzer (Nico2000 Ltd, UK) as a proxy. This data can then be used to determine the kinetics of enamel demineralization before and after each treatment, and at each pH, as previously described [17].

Sound premolars were coated with fluoride-free varnish (Inglot, Poland) leaving a 2 × 2 mm window on the mid-labial surfaces. The 54 teeth were randomly allocated into 18 groups of 3 teeth, with 3 groups allocated to each of the 6 treatment solutions; phosphate buffered saline (PBS) as negative control; 1% and 2% PGGA; and 0.01%, 0.1% and 0.5% NaF (as positive controls). The teeth were immersed in the respective treatment solution for 24 h, and subsequently washed with deionized water to remove excess treatment solution. The teeth from each treatment solution were then immersed in 40 mL demineralizing solution at either pH 4.0, 4.5, or 5.0 at 37 °C, so that there were 3 teeth for each pH value for each of the 6 treatment solutions. A Ca^2+^ ISE was used to measure the increase in calcium ion concentration in the demineralizing solution every minute for 24 h. The rate of calcium ion (R_Ca_^2+^) release from the teeth for each treatment at each pH was calculated from plots of calcium ion release as a function of time using linear regression as previously described [17]. The 24 h time period was selected as long enough for linearity of calcium release to be detected, but not of sufficient duration that the increase in calcium ion concentration in would not be linear due to the gradual accumulation of calcium, *i.e.* that the calcium ion concentrations in each 40 mL beaker were sufficiently below the HAp saturation concentration which is about 30 mmol/L for enamel at pH 4.0 as previously reported [18].

### Determination of the Cross-sectional Micro Hardness (CSMH) of Demineralized Enamel

The teeth used in each Ca^2+^ ISE experiment were then washed in deionized distilled water. Each tooth was cut longitudinally into halves through the developed lesion using a rotating diamond cutter (Micracut 125 Low Speed Precision Cutter, Turkey).

The sectioned teeth were then mounted on epoxy resin leaving the cut section area exposed for microhardness measurement. CSMH measurements were carried out using a microhardness tester with a Knoop indenter (MV-2000 Microhardness Tester, Japan). Indents were made from the outer enamel surface to a depth of 250 µm at intervals of 50 µm (5 depth levels) in both the varnished and unvarnished areas. The indents were made with the cross-sectioned surface perpendicular to the load direction. Five indents were made at each depth with a force of 25 g and a dwell time of 5 s. The KHN was converted to volume percentage mineral via the empirical formula developed by Featherstone et al*.* [19]. The volume percent mineral content at each subsurface depth of enamel was then determined for each treatment group at each pH:

$$Volume Percentage Mineral =4.3 \times (\sqrt{KHN} )+11.3$$

### Statistical Analysis

All data were analyzed using IBM SPSS software version 23 (Chicago, U.S.A). The statistical significance threshold was set at ρ ≤ 0.05. All data were analyzed using a one-way Analysis of Variance (ANOVA) with Bonferroni post-hoc tests.

## Results

### Real-time Ca^2+^ ISE Release

**Fig. 2:**
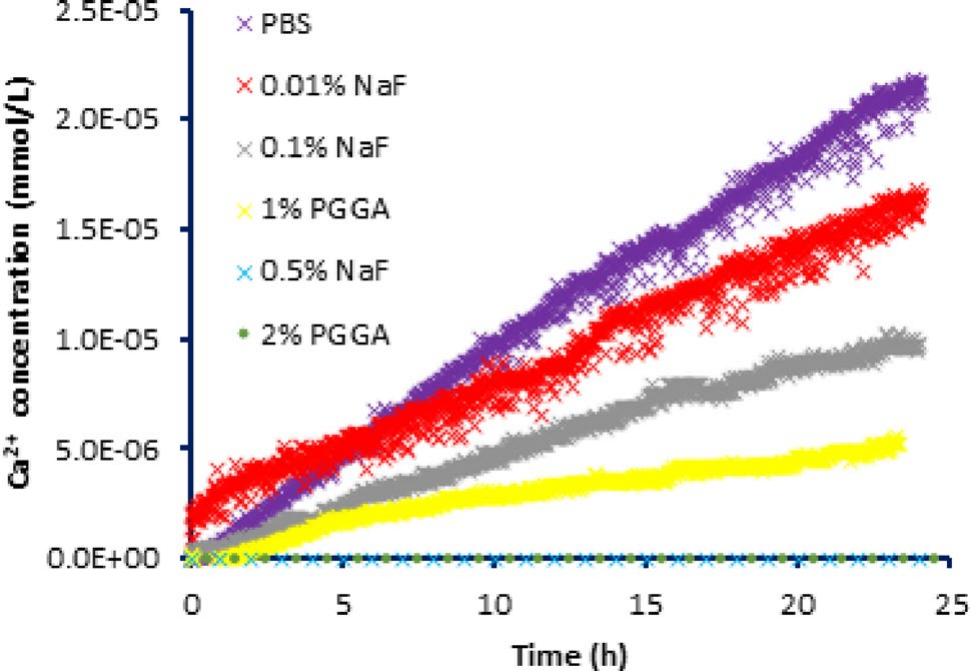
The change in Ca^2+^ concentration following release from enamel *pretreated* with PBS (negative control), 0.01% NaF, 0.1% NaF, 0.5%NaF (positive controls), and PGGA at concentrations 1% and 2%, respectively, upon exposure to 0.1 mol/L acetic acid at pH 5.0, measured at intervals of 1 min and monitored for 24 h. The experiment was carried out in triplicate. The plot shows the average values of Ca^2+^ concentration as a function of time for each treatment. The calcium release was below the detection limit of the ISE for the 0.5% NaF and the 2% PGGA treatments

Figure 2 shows the mean Ca^2+^ ISE measured calcium ion release as a function of time, at 1 min intervals for 24 h, for enamel demineralized at pH 5.0 following each treatment.

Similar plots of the calcium ion release for each treatment were obtained at pH 4.0, 4.5, and 5.0. The plots were then used to calculate R_Ca_^2+^ for each treatment at each pH, and are shown in Table 1.

**Table 1:** R_Ca_^2+^ release for the demineralizing solutions (0.1 M acetic acid at pH 4.0, 4.5, and 5.0, respectively) from the teeth pretreated groups; PBS control (negative control), 0.01%NaF, 0.1% NaF, 0.5% NaF (positive controls), and 1% PGGA and 2% PGGA. The R_Ca2+_ release from pretreated teeth with different treatment groups were significantly different for each treatment (ρ < 0.05) for a given pH value

		Treatment groups					
		PBS	0.01% NaF	0.1% NaF	0.5% NaF	1%PGGA	2%PGGA
R_Ca_^2+^ (mmol/L/h) Mean ± SE	pH 4.0	6.08 × 10^–5^ ± 0.009 × 10^–5^	3.98 × 10^–5^ ± 0.005 × 10^–5^	2.74 × 10^–5^ ± 0.005 × 10^–5^	1.77 × 10^–5^ ± 0.001 × 10^–5^	2.02 × 10^–5^ ± 0.005 × 10^–5^	1.18 × 10^–5^ ± 0.003 × 10^–5^
	pH 4.5	8.66 × 10^–6^ ± 0.038 × 10^–6^	8.07 × 10^–6^ ± 0.009 × 10^–6^	5.46 × 10^–6^ ± 0.081 × 10^–6^	2.66 × 10^–6^ ± 0.005 × 10^–6^	5.41 × 10^–6^ ± 0.015 × 10^–6^	1.03 × 10^–6^ ± 0.003 × 10^–6^
	pH 5.0	9.03 × 10^–7^ ± 0.019 × 10^–7^	5.96 × 10^–7^ ± 0.022 × 10^–7^	4.22 × 10^–7^ ± 0.013 × 10^–7^	-*	2.81 × 10^–7^ ± 0.029 × 10^–7^	-*

^*^Excluded from analysis where there was no calcium ion release

There was calcium ion release at all pH values in the PBS (negative control) group. The percentage inhibition for each NaF and PGGA treatment at each pH was then calculated by comparison of the R_Ca_^2+^ with the data for PBS (negative control) group. This better reflects the efficacy of each treatment, and relates more directly to caries inhibition treatments [17]].

**Fig. 3:**
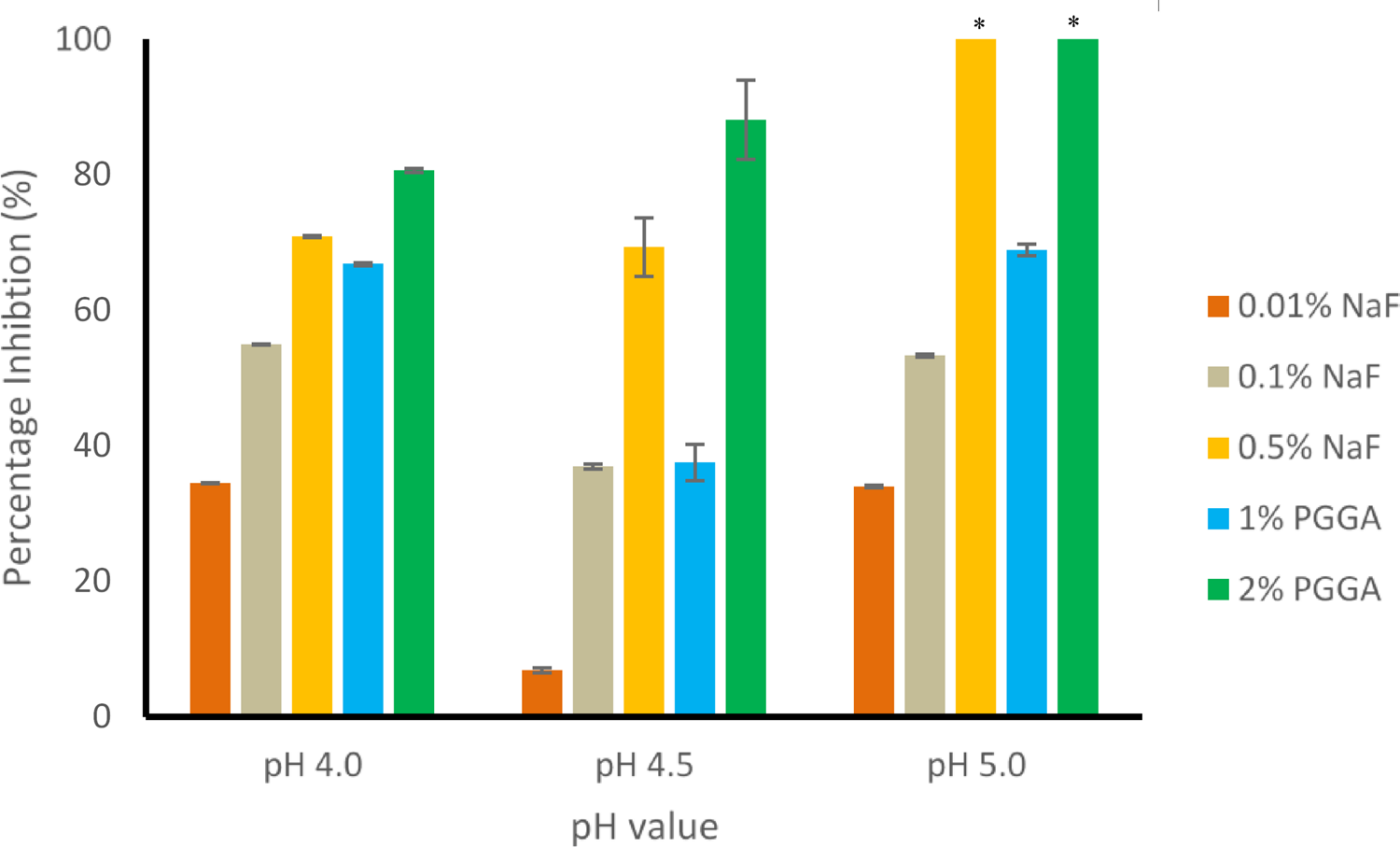
The mean percentage inhibition of calcium ion release from enamel treated with 0.01% NaF, 0.1% NaF, 0.5% NaF (positive controls), and PGGA at concentrations of 1% and 2%, respectively, following exposure to 0.1 mol/L acetic acid at pH 4.0, 4.5, and 5.0. *Excluded from analysis where there was no calcium ion release

Figure 3 shows the percentage inhibition of calcium release for all positive control NaF concentrations, and PGGA treatment conditions at all pH values.

There was an inhibition of calcium ion release seen for enamel pretreated with both PGGA concentrations (and NaF positive controls) at all pH values. The inhibition was greater for the 2% PGGA than 1% PGGA at all pH values. The calcium ion release for 0.5% NaF and 2% PGGA pretreated teeth at pH 5.0 was below the detection limit of the Ca^2+^ ISE even after 24 h, demonstrating that demineralization had been completely inhibited.

A Z-test (p < 0.05) between the treatment groups, including the negative control group (PBS), within the same pHs, showed statistically significant differences in R_Ca_^2+^ release on immersion in demineralizing solution at each pH for each PGGA treatment.

### CSMH of PGGA Pretreated Dental Enamel in Demineralizing Solution (0.1 M Acetic Acid) at pH 4.0, 4.5, and 5.0

**Fig. 4:**
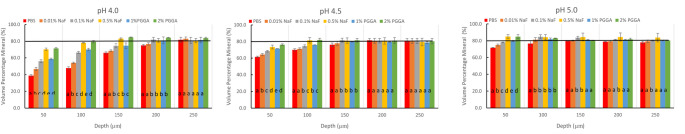
CSMH determined mineral content values of enamel after the effect of demineralizing solution of pH 4.0, 4.5, and 5.0 on pretreated enamel with different concentrations of PGGA compared to PBS and NaF. Mineral Content expressed as Mean ± SD, where the number of determinants (n) is 3. Different lower case letters (**a**–**f**) show statistically significant differences at same depth between different treatment groups at each pH (ρ < 0.05)

Figure 4 shows the enamel mineral content (expressed as percentage volume) at a range of increasing depths from the tooth surface at 50 µm intervals for the different treatment groups.

Statistically significant differences were observed between the control (PBS) and the NaF treatment groups, and between the control (PBS) and the PGGA treatment groups at all depths where demineralization had occurred. The horizontal line shows the mineral concentration values (80%) for unaffected enamel. The enamel treated with 2% PGGA had a greater reduction in mineral content at all depths compared with 1% PGGA, and the untreated control. The effect of the 2% PGGA compared to the PBS control was at least similar to that of the 0.5% NaF at all depths. Further at depths of 100 µm – 250 µm, the measurements suggest that there was a small increase in the mineral content above that of the un-demineralized enamel for the 2% PGGA and 0.5% NaF treated enamel. Even at pH 4.5 and pH 5.0, the 2% PGGA had resulted in significant inhibition of demineralization at subsurface depths.

## Discussion

In this study, unlike previously reported [15], enamel was exposed to pH values of 4.0, 4.5, or 5.0 throughout the experimental period. The Ca^2+^ ISE data showed that the rate of calcium ion release from enamel treated with PGGA (and the NaF positive controls) decreased compared to the negative control at all pH values. Note that the calcium ion release was linear with respect to time for all treatments at all pH values. That the rates are linear with time in the period observed (24 h), for both NaF and PGGA treatments demonstrates (that as previously shown for fluoride ion inhibition of enamel demineralization [20], and other inhibition studies), there is no accumulation effect with time for PGGA over the 24 h period. This suggests that once PGGA is bound to the hydroxyapatite mineral in enamel, there is no further change in binding with time. Note that in those cases where calcium ion release was above the detection limit of the Ca^2+^ ISE, calcium release was detectable even after 1 min. The pattern of decreasing demineralization in enamel with increasing PGGA concentration is in agreement with that previously reported using HAp pellets as enamel analogues [16]. This demonstrates that the process of enamel demineralization inhibition by PGGA, and the inhibition of the demineralization of HAp pellets, is possibly similar to that of statherin, as both PGGA and statherin contain glutamic acid residues that can bind to hydroxyapatite, and, transport calcium ions. Note that at pH 5.0, there was no calcium ion release observed from the enamel pre-treated with 2% PGGA or 0.5% NaF positive control.

The CSMH profile data corroborate the Ca^2 +^ ISE data, showing that enamel treated with 1% and 2% PGGA immersed in demineralizing solution also exhibits significant inhibition of demineralization at all pH values. However, the mineral content profiles as a function of depth demonstrate that the demineralization inhibition is not only a surface phenomenon, but also occurs at greater depths within the lesion. This suggests that PGGA is able to penetrate from the surface to deeper into the lesion. Further, at pH 4.0 for the PGGA, at subsurface lesion depths, there is more mineral than originally present, suggesting that the PGGA that is penetrating deeper into the lesion may also carry calcium ions removed from the surface region into deeper within the lesion. This would need further study to investigate the mechanism of this transport. This increase in mineral content was also seen for the 0.5% NaF treatment at all pHs, though it may be due to an increase in microhardness from fluorapatite formation.

The PGGA results described here, and the preceding PGGA HAp caries and enamel inhibition studies [13–16], suggest that PGGA should be considered as a suitable treatment option for both caries prevention, and caries treatment. It is likely that the *modus operandi* of PGGA is similar to that of the salivary protein statherin as both contain glutamic acid residues.

The delivery method for therapeutically useful PGGA requires further consideration. Further, the interaction of fluoride on PGGA will also need additional investigation. In addition, further studies are also needed to understand the remineralization capabilities of PGGA.

## Conclusions

Ca^2+^ ISE and CSMH studies both show that PGGA reduces the rate of enamel demineralization under caries-like conditions similar to that reported for hydroxyapatite pellets. Further, the mechanism is enhanced by the calcium ion transporting mechanism of PGGA. In addition, the mode of action of PGGA may possibly be similar to the mechanism of the salivary protein statherin.

## Supplementary Information

Below is the link to the electronic supplementary material.

Supplementary Material 1
